# Supervised learning from human performance at the computationally hard problem of optimal traffic signal control on a network of junctions

**DOI:** 10.1098/rsos.140211

**Published:** 2014-12-24

**Authors:** Simon Box

**Affiliations:** Faculty of Engineering and the Environment, University of Southampton, Highfield Campus, University Road, Southampton SO17 1BJ, UK

**Keywords:** traffic control, machine learning, human problem solving

## Abstract

Optimal switching of traffic lights on a network of junctions is a computationally intractable problem. In this research, road traffic networks containing signallized junctions are simulated. A computer game interface is used to enable a human ‘player’ to control the traffic light settings on the junctions within the simulation. A supervised learning approach, based on simple neural network classifiers can be used to capture human player's strategies in the game and thus develop a human-trained machine control (HuTMaC) system that approaches human levels of performance. Experiments conducted within the simulation compare the performance of HuTMaC to two well-established traffic-responsive control systems that are widely deployed in the developed world and also to a temporal difference learning-based control method. In all experiments, HuTMaC outperforms the other control methods in terms of average delay and variance over delay. The conclusion is that these results add weight to the suggestion that HuTMaC may be a viable alternative, or supplemental method, to approximate optimization for some practical engineering control problems where the optimal strategy is computationally intractable.

## 2. Introduction

The set of tasks at which humans can outperform machines has been steadily shrinking. This progress has been punctuated by landmark events where a machine is shown to be able to match or exceed human performance at a task that was previously *only* routinely performed by humans; for example, driving a car in urban traffic [1], playing backgammon [2] or competing in a television quiz show [3]. In each of these examples (and many others), the machine performance was achieved, at least in part, using supervised learning from expert human performance.

There is another class of tasks, which are currently routinely performed by machines but are computationally *hard*. Consequently, the machine strategies for these tasks are arrived at by approximate optimization. Humans may be able to perform well at these tasks but it is impractical or unsafe for them to do so on a regular basis. However, there may be a practical way to employ supervised learning from expert human performance as a method to improve machine performance at these tasks. This paper proposes that this is the case for the task of traffic light signal control on a network of road junctions.

### 2.1 Humans and hard problems

Biological life is known to be able to find near optimal strategies to the solution of problems where there is an evolutionary advantage to high performance. For example, Krebs *et al.* [4] showed that great tits find optimal exploration versus exploitation strategies when foraging, and Tero *et al.* [5] showed that slime mould can construct near optimal networks for nutrient transfer between discrete locations.

Humans, in particular, are capable of exhibiting high performance on some computationally hard problems, including problems where the evolutionary advantage is less clear. For example, some children's computer games are known to be non-deterministic polynomial-time hard (NP-hard) [6]. In this case, perhaps the games share analogous features to activities for which humans have evolved good strategies. Another example is the famous travelling salesman problem (TSP), which is NP-complete [7]. There has been much investigation of human performance on the TSP [8–12] and while modern heuristic graph search algorithms can produce tours on networks with billions of nodes, on limited node networks human performance can come close to the best graph search algorithms. In some earlier works, for example, Michie's study [8], human subjects occasionally beat the leading graph search algorithms of the time.

Traffic light junction control is an optimal switching problem. Unfortunately, optimal switching on a network of interacting road junctions is not achieved by optimal switching on each junction individually [13]. Optimal switching on a network of junctions is known to be computationally intractable, specifically deterministic exponential-time complete (EXPTIME-complete) [14]. Hence, existing traffic light control strategies are all based on approximate optimizations.

### 2.2 Human junction control

The performance of humans at the junction control task has not been widely discussed in existing literature. Human traffic ‘conductors’ are still common in some countries, e.g. North Korea, but in most of the developed world they have largely been replaced by automated systems. Despite this, there have been remarkably few before and after studies on performance of traffic networks that have switched from human control to automated control. Quinn *et al.* [15] present the only systematic analysis that the author is aware of. They document experiments recording the traffic conditions in Bangkok for two consecutive weeks. The first of which the traffic was under police control the second of which the traffic was under automatic traffic control, employing the TRANSYT [16] system. Using measurements of throughput, delay^1^ and average speed [15] showed that in all but a small number of scenarios police control outperformed the automated control system on all measures.

Despite this result, the automated system was viewed as a success and retained in Bangkok. This perhaps points to the reason that before and after studies are rare: performance is not the main motivation for machine-based traffic light control. A key motivator is safety, in part, the safety of drivers and also the safety of the human controllers. There is very clear evidence that police traffic conductors are exposed to dangerous levels of pollutants [17] that lead to negative health effects [18]. Labour costs and usage are also a significant motivating factor. Quinn *et al.* [15] cite the fact that Bangkok police were released from traffic control duties to perform other tasks as a key success of the scheme.

While there is a lack of data from historical studies, this proposition has also rarely been tested in experiment. However, in one embodied simulation experiment carried out by Box *et al.* [19], 30 vehicles with volunteer drivers drove around a test track with figure of eight topology and a traffic light junction at the crossover. In one 15 min test the traffic lights were switched remotely by a novice human controller who was close to the junction in an elevated position 5 m above the road surface. The average time delay (see footnote 1) experienced by vehicles during this test was 30% lower than in an equivalent benchmark test where fixed time control was used. While this may suggest the potential for good human performance, this was an isolated test and did not compare human performance against the kind of traffic-responsive control systems that are used on modern junctions.

### 2.3 Computer game evaluation

While embodied simulation experiments like this can closely model a junction, they do not have perfect fidelity [19] and they require significant resources to perform. A more practical proposition for the first evaluation of junction control strategies is to use computer simulation—specifically a *traffic microsimulation*, which models the individual accelerations of vehicles on a network.

This paper presents a microsimulation-based evaluation of a number of traffic-responsive signal control systems. As benchmark controllers, we employ two systems that are widely deployed in cities today: the MOVA system [20] and the SCOOT system [21]; as well as a temporal difference (TD) learning-based system, developed in part by the author [22].

By using the ability of the microsimulation to output a three-dimensional animated visualization of the vehicles on the network and by augmenting the program to provide it with a human user interface, it is possible to create a *computer game*, enabling the signal control decisions in the simulation to be made by a human *player*.

The approach of using supervised learning to capture a player's strategies from this signal control computer game was first laid out in Box & Waterson [23]. Here the method is extended. Patterns from a human subject's strategy are captured in the same data sources used by MOVA and SCOOT and TD control, leading to the development of a human-trained machine control (HuTMaC) system with performance that compares favourably against MOVA and SCOOT and TD control.

The scope of this paper is to investigate the use of supervised learning to capture the performance of a single human subject known to be a good player of the game (see appendix C for some data on the relative performance of our subject). In other work, the same computer game is being used to investigate the variation in performance between human subjects and to analyse the strategies that they employ. That work is reported separately [24].

## 3. The microsimulation

### 3.1 Simulation environment

The work described here used the Paramics microsimulation environment described in [25,26]. Data on the efficacy of the Paramics simulator have been reported in a number of validation and calibration studies, e.g. [27–30].

**Figure 1.**
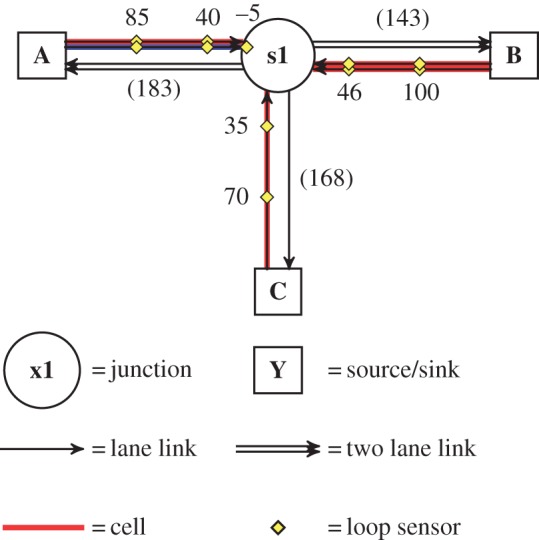
Topology of the T-junction model. Numbers in parentheses indicate the length of the link in metres. Numbers next to loop sensors indicate the location of the sensor, measured in metres from the downstream end of the link. The links that are highlighted are also *cells* for use in the *cell-based state* (see §5.2.2).

**Figure 2.**
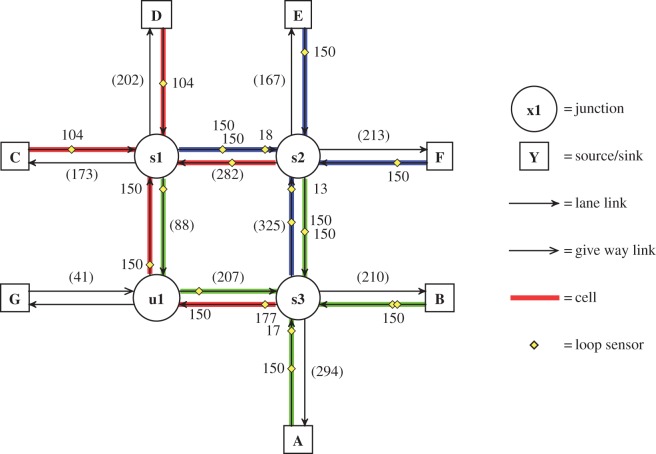
Topology of the Multi-junction model. Numbers in parentheses indicate the length of the link in metres. Numbers next to loop sensors indicate the location of the sensor, measured in metres from the downstream end of the link. The links that are highlighted are also *cells* for use in the *cell-based state* (see §5.2.2).

Two road network models were used. MOVA, which is designed to operate on isolated junctions, was evaluated on the T-junction model (figure 1). SCOOT, which is designed to operate on small networks of junctions (and coordinate the action between them) was evaluated on the Multi-junction model (figure 2). These two models were developed by the Transport Research Laboratory (TRL)^2^ and Siemens PLC^3^ as exemplar networks for (respectively) MOVA and SCOOT control. TRL and Siemens also provided the author with validated control programs for these models and the MOVA and SCOOT systems. The TD control system, human control and HuTMaC control were all evaluated on both the T-junction and Multi-junction models.

**Figure 3.**
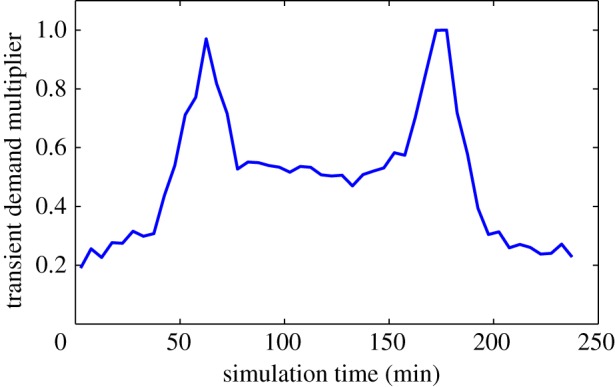
Variation in the demand multiplier *γ*_*t*_ over the 4 h testing time.

The number of vehicles in the microsimulations is determined by the in-flow rate at the source/sink nodes in the network model, which are labelled (A-G) in figures 1 and 2. The baseline vehicle in-flow rate for trips between each source/sink node are given in appendix A. During a simulation experiment, the actual vehicle in-flow rate is calculated as a product of the baseline rate and a *demand multiplier* (2.1):

3.1 $$S_{o,d}=s_{o,d}\gamma_{t},$$

where *S*_*o*,*d*_ is the actual in-flow rate of vehicles at origin node o, travelling to destination node d, *s*_*o*,*d*_ is the baseline rate and *γ*_*t*_ is the demand multiplier. The demand multiplier may be fixed for the duration of the test or it may be transient, varying over the time of the test according to a specified function, for example, the function shown in figure 3. In implementation, the generation of new simulated vehicles at source nodes is a stochastic process and *S*_*o*,*d*_ represents the average rate over time.

### 3.2 Signal control application programming interface

An application programming interface (API) was built to allow external signal control programs to connect to the microsimulation and control the traffic lights. A signallized junction is a finite state machine. Sets of turning movements that do not conflict are grouped together into *stages* and each junction can be set to only one stage at a time. The precise assignments of turning movements to stages for each signallized junction node in figures 1 and 2 are given in appendix A.

The junction control strategy is a policy for switching between stages, bearing in mind that there is a switching penalty imposed by the fact that there is a brief (usually around 7 s) period during the switch between stages when the lights are either amber or all red, this is known as the inter-green time. The inter-green time for each stage transition is fixed in the network model and the signal control programs simply call for a stage, which triggers the switching process.

Signal controllers must be able to perceive the traffic state and respond accordingly. In practice, perception data are obtained from sensors within the traffic network. One of the most commonly used sensors is the *inductive loop*. These are metal detectors buried in the surface of the road. The detectors return a binary signal at 4 Hz indicating the presence or absence of metal above the loop. Data such as *count, flow* and *occupancy* (see §5.2.1) of vehicles over the loop can be inferred from this signal.

Inductive loops are simulated within the Paramics environment. The locations of these sensors within the T-junction and Multi-junction models are indicated in figures 1 and 2. The API can serve connected signal controllers with data from the simulated inductive loop sensors. In addition to the loop data, the API can also serve data on the instantaneous positions and speeds of all vehicles within the simulation. The ways that these data are used by the various (non-human) signal control systems in this paper are described in §§4 and 5. The human controller uses a different API altogether, the *computer game interface*, which is described in the following section.

### 3.3 Computer game interface

**Figure 4.**
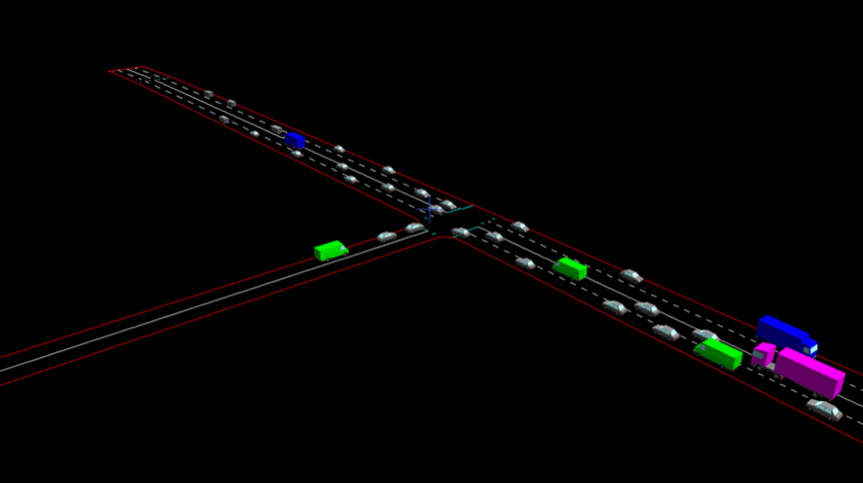
Screen shot from the computer game running the T-junction model.

To enable human control of the junctions in the T-junction and Multi-junction models, Paramics' ability to output a three-dimensional animated visualization was used. The player of the game has an elevated view of the junction network (figure 4), which can be panned and zoomed if necessary. The player can watch the individual vehicles in the simulation driving through the network. The player can also choose the speed at which the simulation runs. Most players choose to play at four times faster than real time. In gameplay, the simulation runs for a (simulated) time of 10 s (2.5 s in player time) and then pauses and prompts the player to select a signal stage. The player does this by inputting a number on the computer keyboard that corresponds to the stage. If the network contains more than one junction the player is prompted to make multiple decisions like this, one for each junction.

If the player picks the stage that is currently active then the simulation continues with no change, if the player selects a different stage then a switching operation is triggered. This approach ensures that decisions to ‘stick with the same stage’ are recorded in the same way as decisions to switch stages, which is advantageous for generating the training data for supervised learning. A limitation of this approach is that the player can *only* make a decision every 10 s, where as other control systems (e.g. MOVA and SCOOT) can call for stage changes at a precise second.

### 3.4 Evaluation measurements

To evaluate the performance of the control strategies employed in the simulation experiments, the *delay* measure is used. For a given simulated vehicle *p*, the time it takes to travel from its origin o to its destination d is its journey time *δ*_*p*_. The vehicle's free flow travel time *δ*^(ff)^_*o*,*d*_ is the theoretical average time that it would take to travel between o and d if *p* were unimpeded by other vehicles or red signals. The delay for vehicle *p* is the difference between these two times (2.2):

3.2 $$\theta_{p}=\delta_{p}-\delta_{o,d}^{\left(\mathrm{ff}\right)}.$$

In this paper, we evaluate the performance of signal control strategies by looking at both mean delay—*μ*(*θ*) and also standard deviation over delay—*σ*(*θ*), which is a good proxy for how equitable the treatment of vehicles is under a signal control strategy. The performance goal is to jointly minimize both these measures.

## 4. Benchmark control methods

The computational time required to find the optimal switching policy (to maximize throughput) on a network of queues with stochastic arrival of ‘customers’ grows faster than polynomially with network size, in other words, the problem is EXPTIME-complete [14]. Therefore, modern traffic-responsive signal control systems are based on approximate optimization. In this paper, the performance of HuTMaC is compared with three benchmark approximate optimization-based systems. The MOVA [20] and the SCOOT [21] systems are in wide use today [31] and use inductive loop measurements to inform signal control decisions. TD control [22] is applied using measurements of individual vehicle's position and speed—albeit in a compressed form. This is to provide an example of how approximate optimization methods may perform with high quality traffic state data. In this section, the set-up of MOVA and SCOOT and TD control are described. The supervised learning approach of HuTMaC is described in the following §5.

### 4.1 MOVA and SCOOT control

The precise algorithms employed by MOVA [20] and SCOOT [21] are proprietary and unknown to the author, however, industrial partners the TRL^4^ and Siemens PLC^5^ provided this project with instances of the MOVA and SCOOT systems and API's to allow those instances to be connected to the Paramics microsimulation. The T-junction and Multi-junction models were also provided as exemplars for, respectively, MOVA and SCOOT control and the positions of the inductive loop sensors in these models were determined by the requirements for MOVA and SCOOT. The inductive loop sensors provide the input *state* data for the MOVA and SCOOT systems.

### 4.2 Temporal difference control

As widely deployed systems, MOVA and SCOOT are useful benchmarks. However, many other approximate optimization-based control systems have been proposed in the research literature more recently and—while they have not been adopted (yet)—many demonstrate high performance. Several of the approaches proposed in research also consider the potential use of richer sources of traffic state data such as GPS probes within vehicles reporting position and speed. One particular approach that is currently popular in the signal control research literature is TD learning [32–37]. TD learning in the form applied in these papers shares many properties with approximate dynamic programming. It essentially arrives at strategies by exploring a discrete state-action space and then using feedback on performance to adjust a nominal ‘value’ of recently visited state-action combinations, thus tuning the control strategy.

In fact, the discrete TD learning approaches in [32–37] can be extended to a continuous state action space by employing a function approximator, e.g. a neural network. This has been demonstrated by Tesauro [38] in application to computer backgammon programs, and more recently by the author [22] in application to traffic signal control.

This type of TD control is attractive as a benchmark against which to measure HuTMaC control. This is because the HuTMaC supervised learning procedure also employs a neural network to classify the state space (see §5). Thus, it is possible to use an identical neural network structure, including identical input data for both TD control and HuTMaC. This means that the method by which the parameters of the neural network are tuned is the *only* difference between the two approaches. That is the approach followed in this paper and it is one that provides relatively pure comparison between approximate optimization and supervised learning from a human, because the precise strategy which is arrived at by HuTMaC is also available to TD learning and vice versa.

The TD control system as described in [22] was employed. As in [22], a compressed form of individual vehicle position and speed data are used to describe the control state space. This state space is described in detail in the context of HuTMaC in §5.2.2. The optimization (training) procedure described in [22] was also followed. In this case, the T-junction and Multi-junction models used 260 h and 780 h of microsimulation time respectively, to achieve the performance presented in the results in §6.

## 5. Human-trained machine control

The approach of HuTMaC is to apply supervised learning to data collected when a human player controls a simulated traffic network model via the computer game interface, as described in §3.3.

### 5.1 Supervised learning

Control of a finite state machine can be equivalent to a classification problem. A state space describing the conditions on the road network must be classified into regions corresponding to junction stages. Thus, a transition between two regions of state space triggers a transition between the two corresponding junction stages. The training data for the classification are generated by the human computer game player. The classifier employed in this paper is a two layer neural network of the type in (4.1):

5.1 $$\text{y}=f({\text{W}}^{\left(2\right)}g({\text{W}}^{\left(1\right)}\text{b})),$$

where *g*(**a**) is the hyperbolic tangent function $g(a_{h})=\tanh(a_{h})$ and *f*(**u**) is the softmax function given by (4.2):

5.2 $$f(u_{k})=\frac{\exp(u_{k})}{\sum_{q=1}^{K}\exp(u_{q})}.$$

In (4.1), **b** is a *J* dimensional vector that describes the current *state* of the road network around the junction (see §5.2). Matrices **W**^(1)^ and **W**^(2)^ are the neural network's parameters. **W**^(1)^ has dimensions *J*×*H*, **W**^(2)^ has dimensions *H*×*K*, where *K* is the junction's number of signal stages. *H* is the number of *hidden units*. The output vector **y** contains *K* elements, one for each stage of the junction being controlled:

5.3 $$\sum_{k=1}^{K}y_{k}=1.$$

A neural network of type (4.1) is associated with each junction in the road network under HuTMaC. Every time the player presses a key to make a stage decision a new pattern is added to the training data. Each pattern *n* in the set of *N* patterns consists of a state **b**_*n*_ and a corresponding decision vector ***τ***_*n*_ with elements

5.4 $$\tau_{k}\in \{0,1\},$$

and the condition

5.5 $$\sum_{k=1}^{K}\tau_{k}=1.$$

When the *k*th element of ***τ***_*n*_ is 1, this indicates that the decision was to select junction stage *k*.

The parameters **W** are learned from the training data using the following numerical procedure: **W** is initialized randomly and cross-entropy error (4.6) is calculated for each pattern in the training data:

5.6 $$E_{n}=-\tau_{n}{\left(\ln({\text{y}}_{\text{n}})\right)}^{T}.$$

The total error is summed over all *N* patterns $E=\sum_{n=1}^{N}E_{n}$. The parameters are updated using the gradient of the error function in parameter space:

5.7 W←W−η∇E(W),

where the coefficient *η* is the *learning rate*. The gradient of the error function with respect to the network parameters is calculated using (4.8) for the first layer parameters **W**^(1)^ and (4.9) for the second layer parameters **W**^(2)^:

5.8 $$\nabla E{\left({\text{W}}^{\left(1\right)})=\text{b}(\mathrm{diag}(1-z_{h}^{2}){\text{W}}^{\left(2\right)}(\text{y}-\tau )\right)}^{T}$$

and

5.9 $$\nabla E({\text{W}}^{\left(2\right)})=\text{z}{\left(\text{y}-\tau \right)}^{T},$$

where $\text{z}=\tanh({{\text{W}}^{\left(1\right)}}^{T}\text{b})$.

This continues iteratively until a local minimum is found. The entire process is repeated 30 times, each with different random initializations of **W**, to avoid a result in a poor local minimum.

The parameters with the lowest final error are selected and neural networks (4.1) at each signallized junction node, with these tuned parameters, are the HuTMaC controllers. In operation the HuTMaC controller uses a 10 s time step, sampling the state and selecting the appropriate junction stage at each step.

### 5.2 Description of the traffic state

We have described how the HuTMaC system achieves junction control through a classification of state space. We now turn to the structure of the state space and the data that make up the state vector **b**. Here the motivation is to use states that employ equivalent data to the benchmark control methods used, namely MOVA, SCOOT and TD control. MOVA and SCOOT both use inductive loop data to describe the traffic state. So for comparison with MOVA and SCOOT, HuTMaC uses an inductive loop-based state that is described in §5.2.1 below. The TD controller described in [22] uses a compressed form of individual vehicle position and speed data called the cell-based state. So for comparison with TD control, HuTMaC uses the same cell-based state which is described in §5.2.2 below.

#### 5.2.1 Inductive loop-based state

The signal from an inductive loop sensor can be processed to obtain data such as *count*, which is the number of vehicles that cross a loop in a given time period *C*(Δ*t*); or *occupancy*, which is the fraction of time for which presence is detected in a given time period *ρ*(Δ*t*).

To construct the state vector at a given time step **b**_*t*_, *L* loop sensors in the vicinity of the junction are selected and the occupancy of each loop sensor *l* over the 20 s prior to the time step is placed in **b**_*t*_ (4.10). Also added to **b**_*t*_ are the stage decisions for the previous two time steps (4.10):

5.10 $${\text{b}}_{t}=[\rho_{t,1}(20),\ldots ,\rho_{t,L}(20),k_{t-1}^{\prime },k_{t-2}^{\prime },1],$$

where $k_{t}^{\prime }$ indicates the value of *k* for which *τ*_*k*_=1 at time *t*. Finally, following the convention for neural network inputs, a unit offset element is appended to **b**_*t*_ so the total number of elements in **b**_*t*_ is *L*+3.

#### 5.2.2 Cell-based state

The TD controller described in [22] uses data on the position and speed of individual vehicles within the simulation. While this provides a much richer source of information than the inductive loop sensors it has the drawback that the raw position and speed data have high dimensionality. Both TD control and HuTMaC involve fitting functions to the state space, so limiting the dimensionality is computationally advantageous. Thus, in [22] and in this paper the data are compressed in the following way.

Following the cell transmission modelling approach of [39], the road network is divided up into small regions (*cells*) and data are attached to each cell, for example, a count of the number of vehicles a cell *i* (*C*_*i*_), or the average speed of vehicles in a cell (${\bar{V}}_{i}$). Within this framework it is possible to vary the number (*I*) and size of cells as well as the number (*M*) of data types attached to each cell, thus varying the dimensionality (*I*×*M*) of state space.

Following the approach in [22] the T-junction and Multi-junction network models are coarsely divided into cells that cover a whole link. The links which are used as cells are highlighted in figures 1 and 2. The one exception is link A:s1 in figure 1. This link is divided into two cells—one for each lane—as indicated by the different colour and shade of highlighting. This is so that right turning vehicles waiting in the right hand lane will be detected explicitly.

Each cell *i* has a single metric *b*_*i*_ associated with it, calculated as follows:

5.11 $$b_{i}=\sum_{p=1}^{P_{i}}1-\alpha V_{p}-\beta X_{p},$$

where *P*_*i*_ is the number of vehicles in cell *i*. *V*_*p*_ is vehicle speed and *X*_*p*_ is the distance of the vehicle from the next downstream junction stop line. *α* and *β* are coefficients that determine the relative influence that $\bar{V}$, $\bar{X}$ and *P* each has on the size of *b*_*i*_. *α* and *β* are not ‘tuned’ but are assigned order of magnitude values of *α*=0.01 sm^−1^ and *β*=0.001 m^−1^ to ensure that no term dominates *b*_*i*_ simply by virtue of the units used. For example, distance from the stop line in metres will often be a much larger number than speed in metres per second. This metric is not designed or intended to model any particular aspect of the traffic state, it is simply a way of encoding the information in each cell into a single dimension.

To construct the state vector at a given time step **b**_*t*_ *I* cells within the vicinity of the junction are selected and the metrics *b*_*i*_ are placed in **b**_*t*_:

5.12 $${\text{b}}_{t}=[b_{t,1},\ldots ,b_{t,I},1].$$

These are the only data that are added to **b**_*t*_, in contrast to the loop-based state (§5.2.1) which also added previous stage decisions in (4.10).

In simulation, it is possible to record these data with perfect accuracy. Of course, in practice these data would have to be measured, e.g. using GPS and reported, e.g. using WiFi. While this is feasible, the estimates of *P*, $\bar{V}$ and $\bar{X}$ will be subject to noise and errors. For further discussion on the design of this state space representation including results from experiments where noise and errors in measured data are simulated, see [22]. In this paper, we are employing this method as an *upper benchmark* and perfect input data are assumed.

### 5.3 Training procedure

In order to generate the training data for HuTMaC control the human subject was asked to play six games, each lasting for 30 (simulated) minutes, with a minimum break of 30 real time minutes in-between. The inclusion of the breaks was intended to minimize the effects of player fatigue. During each game the in-flow rate was constant, but it was raised between games by adjusting the demand multiplier. The values used were *γ*_*t*_=[0.4,0.6,0.8,1.0,1.2]. This training procedure was performed on both the T-junction and Multi-junction models while stage decision data were recorded, as were inductive loop data and cell metric data. The training data were then used to learn the parameters of the neural networks associated with each of the signallized junctions in the T-junction and Multi-junction models, as described in §5. The precise input data and neural network structure and number of training patterns *N* for each signallized junction node in the T-junction and Multi-junction models are given in appendix B.

## 6. Simulation experiments

Using the microsimulation platform described in §3 experiments were conducted on the benchmark control methods described in §4 and on the HuTMaC system described in §5. Table 1 shows the configurations of control systems and road network models that were tested. For each row in table 1, 10 independent simulation experiments were performed, with each single experiment being a simulation with a duration of four (simulated) hours. The in-flow rate of vehicles throughout the tests was varied according to the demand multiplier function shown in figure 3.

**Table 1.** Summary of the main statistics for each of the configurations tested in the simulation experiments. (Each statistic is calculated over 10 independent runs of the simulation experiment and the statistics are mean delay (*μ*(*θ*)) and standard deviation over delay (*σ*(*θ*)). *p*-values for two-sample *t*-tests between selected control systems are also shown.)

	statistics		*p*-value	
	*μ*(*θ*)(s)	*σ*(*θ*)(s)	(*μ*)	(*σ*)
T-junction				
MOVA	23.95	48.66	3.80×10^−2^	2.16×10^−3^
HuTMaC(loops)	21.51	35.98		
TD(cells)	23.16	40.07	1.89×10^−7^	2.97×10^−4^
HuTMaC(cells)	18.34	32.27		
Multi-junction				
SCOOT	49.76	37.83	1.50×10^−14^	2.31×10^−5^
HuTMaC(loops)	33.31	29.70		
TD(cells)	27.20	20.30	2.87×10^−9^	7.92×10^−3^
HuTMaC(cells)	25.44	19.42		

The statistics of mean delay (*μ*(*θ*)) and standard deviation over delay (*σ*(*θ*)) that are presented in table 1 were calculated over all 10 independent simulation experiments. The detailed statistics for each individual experiment are presented in a much larger table (table 8) in appendix D. The rows of table 1 are organized such that each row for HuTMaC is located directly below the row for the appropriate benchmark control system. For example, for HuTMaC using the loop-based state the appropriate benchmark is MOVA or SCOOT, depending on which road network model is being used. In each case HuTMaC's statistics for mean delay—*μ*(*θ*) and standard deviation over delay—*σ*(*θ*) are lower than that of the benchmark tests. The statistical significance of these performance differences was analysed using two-sample *t*-tests, where the null hypothesis is that the average values of the statistics over the 10 independent tests for each of the two control systems being compared is the same. The *p*-values are given in the last two columns of table 1.

### 6.1 Visualization of results

**Figure 5.**
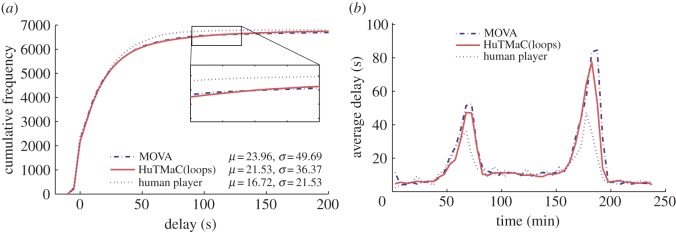
Results from experiments on the T-junction model using a loop-based state. Statistics are calculated over all completed trips during the 4 h testing period. (*a*) The cumulative distributions over vehicle delay, with mean and standard deviation statistics in the legend. (*b*) Transient delay, averaged over 5 min periods throughout the duration of the tests.

**Figure 6.**
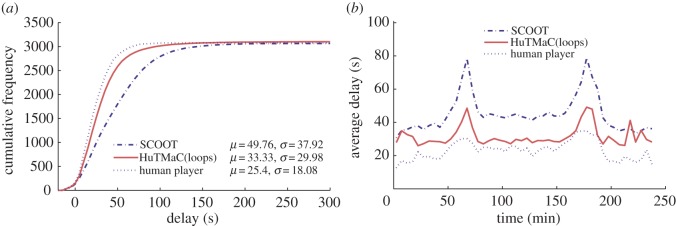
Results from experiments on the Multi-junction model using a loop-based state. Statistics are calculated over all completed trips during the 4 h testing period. (*a*) The cumulative distributions over vehicle delay, with mean and standard deviation statistics in the legend. (*b*) Transient delay, averaged over 5 min periods throughout the duration of the tests.

**Figure 7.**
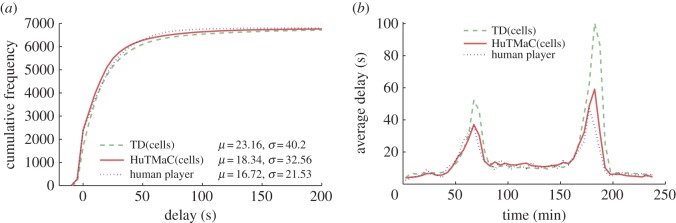
Results from experiments on the T-junction model using the cell-based state. Statistics are calculated over all completed trips during the 4 h testing period. (*a*) The cumulative distributions over vehicle delay, with mean and standard deviation statistics in the legend. (*b*) Transient delay, averaged over 5 min periods throughout the duration of the tests.

**Figure 8.**
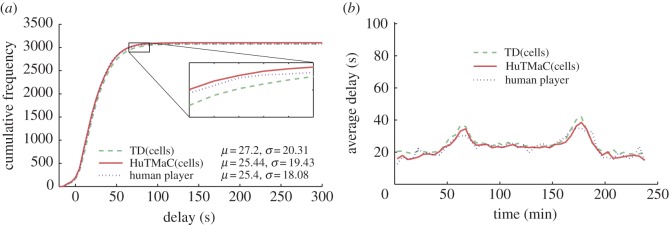
Results from experiments on the Multi-junction model using the cell-based state. Statistics are calculated over all completed trips during the 4 h testing period. (*a*) The cumulative distributions over vehicle delay, with mean and standard deviation statistics in the legend. (*b*) Transient delay, averaged over 5 min periods throughout the duration of the tests.

In this section some visualizations of the results summarized in table 1 are presented (in figures 5–8) and discussed. For reference these visualizations also show examples of human control. The data for these examples were generated by asking the human subject to use the computer game interface described in §3.3 to control simulations equivalent to those described above. During each 4 h simulation the subject was instructed to play for stretches of 30 simulated minutes (7.5 min of real time), then pause and take a break for a minimum period of 30 real-time minutes. Again, this was an attempt to minimize the effects of fatigue on human performance.

Figure 5 shows the comparison between the HuTMaC system using the loop-based state (HuTMaC(loops)) and MOVA on the T-junction model. In each case, the data are averaged over the 10 independent experiments. The left hand plot shows cumulative distributions over delay. These distributions allow us to visualize both the average delay, on the left of the plot and the variance over delay, particularly, in the tail at the top of the plot. In general, distributions closer to the top-left are preferable. The distributions in figure 5 show little difference between HuTMaC(loops) and MOVA, however, close inspection of the expanded area shows the point where the distributions cross, indicating that HuTMaC(loops) has less vehicle trips exhibiting the highest values of delay. This effect is seen in the statistics where HuTMaC(loops) has lower *σ* than MOVA. It can also be seen in the transient delay plot on the right of figure 5. Here average delay during subsequent 5 min periods of the 4 h testing period are plotted. The variation in delay seen in this plot mirrors the variation in the in-flow rate of vehicles during the simulation shown in figure 3. The plot indicates that the main differences in performance between the control systems occurs in the peaks of heaviest traffic. Both plots in figure 5 show that while HuTMaC(loops) has achieved a performance improvement over MOVA, its performance is lower than the example of human performance plotted, which was generated by the same human subject who trained HuTMaC in this case.

Figure 6 examines the performance of HuTMaC(loops) on the Multi-junction model and compares it to the SCOOT control system. The EXPTIME-complete result discussed in §4 implies that the computational complexity of optimal control on a network of three junctions is much greater than for a single junction. So it is notable that the relative difference in performance between the control systems shown in figure 6 is greater than in figure 5. The data indicate that HuTMaC(loops) has lower delay and variance over delay than SCOOT but not as low as the example of human control. Also, the transient delay plot on the right in figure 6 indicates that the greater difference in performance is in the peaks of heaviest traffic but that there is also a consistent difference in performance throughout the experiments.

Figures 7 and 8 examine the performance of the HuTMaC system using the cell-based state (HuTMaC(cells)) and TD control. These figures show that TD control can exhibit high performance, particularly on the Multi-junction model, but in both cases HuTMaC(cells) has lower delay and variance over delay. Figures 7 and 8 also show that the performance of HuTMaC(cells) comes close to matching the examples of human performance. This implies that the cell-based state, in spite of its lower dimensionality, contains more useful information than the loop-based state, where ‘useful’ means for the purposes of supervised learning from human subject's control strategy.

While controlling the simulation via the computer game interface the human subject is provided with richer information than that which is contained in either the loop-based state or the cell-based state, because they can—in principle—perceive the position and speed of every vehicle through the on-screen animation. This raises a question: how much of the human subject's performance is attributable to the better data they receive and how much is due to their strategy? We can deconvolute this through a comparison between HuTMaC(loops) and HuTMaC(cells). The performance results of HuTMaC(loops) show that—to some extent—the strategy of the human player can be captured in the lower fidelity inductive loop data, leading to performance improvements over MOVA and SCOOT. These improvements can be attributed to the human subject's strategy. The results for HuTMaC(cells) indicate performance very close to that of the human subject and an improvement over TD control. This illustrates how the cell-based state, although compressed relative to the information that the human subject receives, nevertheless, contains sufficient information to capture and reproduce the human subject's strategies.

## 7. Discussion

### 7.1 Comments relating to traffic control

The results in this paper have demonstrated that supervised learning from a human subject's control strategies can enable the development of a HuTMaC system for signallized traffic junctions. The performance of HuTMaC has been demonstrated on two road network models, where it exhibited comparable performance to the benchmark control systems of MOVA, SCOOT and TD control.

The author is not proposing that HuTMaC—as presented in this paper—is a viable replacement for current systems like MOVA and SCOOT, because of several limitations and (as yet) unanswered questions summarized in §7.1.1 below. However, the author does propose that supervised learning from humans, which is neglected in favour of approximate optimization in current traffic control systems, can be a powerful tool and that it is worth investigating how this may be leveraged in the design of future systems. While the HuTMaC system presented here is a purely supervised learning system, it is probable that any practical implementation would be a hybrid system. Some discussion is given to this in §7.1.2 below.

#### 7.1.1 Limitations and unanswered questions

*Scaling to large networks*. In this paper, HuTMaC has been demonstrated on a small network containing three signallized junctions. A problem facing HuTMaC control (and all other junction control systems) is how to scale up the approach to a national road network. In the case of SCOOT this is accomplished by coordinating junctions in discrete sub-networks containing small numbers of junctions, known as SCOOT *regions*. Only high level information is shared between regions [21]. Another approach to consider is using principles of self organization to design control strategies for individual junctions that have coordination as an emergent property at the network level as in [40]. Understanding how self-organization could be applied along with supervised learning is a challenge for future work. However, a similar problem has been explored in multiplayer computer games where humans collaboratively solve graph colouring problems [41].

*Constraints.* MOVA and SCOOT are proprietary systems and it is possible that they are subject to some constraints, perhaps in the name of safety, of which the author is unaware and have therefore not been replicated in HuTMaC control. In this case, it is not clear that HuTMaC control is demonstrably unsafe and if a large fraction of the performance difference is owing to a particular constraint, then perhaps these results should prompt an evaluation of the benefits of that constraint.

#### 7.1.2 Future practical systems

The advantages of the HuTMaC system presented in this paper are primarily its performance but it also has a certain flexibility: it has been demonstrated working with existing sensors and it does not need to prescribe specific sensors at specific locations. The training (set-up) time is also relatively short; 180 min of simulated time (45 min of real time) were used to train the HuTMaC system on each road network model. Nevertheless, there are still concerns around applying a pure supervised learning solution, for example, there are no guarantees regarding the performance of HuTMaC in untrained regions of state space. A pragmatic solution may be a hybrid system that employs a traditional approximate optimization-based controller like SCOOT that is augmented by HuTMaC routines that can be employed in specific scenarios. For example, the results in §6 suggest that HuTMaC is particularly useful in the heaviest traffic scenarios.

### 7.2 Comments relating to control problems in general

The application of HuTMaC to traffic signal control in this paper may be considered as a case study and it may be possible that there are other practical engineering control problems to which a similar approach may be applied. In this section, we discuss some of the results in this paper that have general relevance to the solution of control problems.

#### 7.2.1 Computational complexity

A particular advantage of HuTMaC over approximate optimization may be its computational complexity. Some insight into this can be gained by comparison of the HuTMaC(cells) and TD(cells) systems in this paper. Both approaches (described in §5 and [22], respectively) use similar back-propagation algorithms to tune the parameters of identical neural networks and both have complexity *O*(*NW*^2^), where *W* is the total number of parameters in the neural network and *N* is the number of signal control decisions evaluated.

However, in order to achieve the performance statistics presented in figure 8 HuTMaC needed to evaluate *N*=1080 signal control decisions in 3 h of simulation. By contrast, TD control needed to evaluate *N*=280 800 signal control decisions in 780 h of simulation. Optimizing to match a human strategy is apparently simpler than optimizing the strategy directly. This suggests that supervised learning from a human has a systematic advantage over approximate optimization—in computational terms—as long as the human subject is providing good training data.

#### 7.2.2 Human reliability

The supervised learning approach used in this paper makes no attempt to assess the correctness of human decisions before capturing them. In fact, anecdotally players of the traffic control computer game do make mistakes. A common mistake is to intend to select a given stage and then mistakenly press the wrong button. Of even greater concern than these *random* errors would be systematic erroneous biases in the decision-making of the human controller.

The work of Kahneman and others in psychology has identified many scenarios where human decision-making is systematically biased. For example, *loss aversion bias* describes an effect where the cost that humans assign to a loss is apparently greater than the value that they attach to an equivalent gain [42]. In the context of our traffic control game, the player may perceive the build-up of queues as a ‘loss’ and vehicles passing through the junction without stopping as a ‘gain’. In this case does the player exhibit loss aversion bias?

In general, we cannot say how far the performance of the human (or any other controller) is from the (intractable) globally optimum control strategy, but an analysis of systematic biases would be useful in highlighting where errors may occur.

Finally, for problems where human control is impractical or unsafe, or has simply never even been tried, the computer game approach used here is a useful way to evaluate human performance and capture their strategies. In particular, the computer game environment is very *clean* and allows for fatigue and complicating environmental factors (e.g. weather, distraction) to be controlled, thus extracting a relatively pure example of human performance at a problem.

## Supplementary Material

1. simulationData.zip - This zipped folder contains the simulation configuration files for the traffic microsimulation models used in the paper, as well as the raw data output from the simulations. The data are organised into a folder structure with README.txt files used to provide guidance on the contents and how to use them.

## Figures and Tables

**Figure 9.**
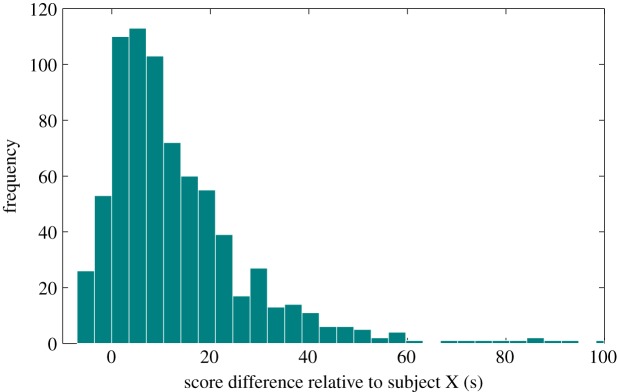
Distribution over players differential score (player's score − our subject's average score). Negative scores outperform our subject, while positive scores underperform our subject.

## Footnotes

1 For a formal definition of delay see §3.4.

2 See http://www.trl.co.uk.

3 See http://www.mobility.siemens.com.

4 See http://www.trl.co.uk.

5 See http://www.mobility.siemens.com.

## Data accessibility

The simulation configuration files and raw simulation data associated with this study are available as electronic supplementary files.

## Appendix A. Microsimulation settings

This appendix contains additional information on the microsimulation settings for the Multi-junction and T-junction models. Tables 2 and 3 show the baseline in-flow rates of vehicles between origin/destination nodes in the T-junction and Multi-junction networks, respectively. The final in-flow rate is calculated using equation (2.1).

**Table 2.** Basic in-flow rate matrix (vehicles per hour) for the T-junction model.

	destination		
	A	B	C
origin			
A	—	1138	300
B	1441	—	76
C	243	243	—

**Table 3.** Basic in-flow rate matrix (vehicles per hour) for the Multi-junction model.

	destination						
	A	B	C	D	E	F	G
origin							
A	0	480	678	192	72	30	96
B	294	0	966	678	36	60	96
C	96	294	0	96	192	1932	96
D	192	192	48	0	12	30	96
E	582	96	294	96	0	96	96
F	384	294	192	48	60	0	96
G	0	0	0	0	0	0	0

Tables 4 and 5 show how vehicle movements are assigned to stages in the signallized junction node s1 of the T-junction model (table 4) and to the three signallized junction nodes s1, s2, s3 of the Multi-junction model (table 5). When switching between stages there is a 7 s inter-green period where the lights are amber or all red. There is also a 2 s delay in the system between calling for a stage and the lights beginning to change. With a 10 s time step (as used in this paper) there is an effective minimum green time of 5 s. There is no maximum green time.

**Table 4.** Showing the assignment of *movements* to junction stages for the junction represented by node s1 in figure 1. (The junction can be in one of three configurations (*stages*). The movements that have the green light during each stage are listed, where X : Y indicates the movement for vehicles travelling between node X and Y, through the junction node. By implication all movements not listed have a red light.)

node	stage	movements
s1	1	A:B,A:C,B:A,B:C
	2	A:B,A:C
	3	C:A,C:B

**Table 5.** Showing the assignment of *movements* to junction stages for each of the signallized junction nodes (s1, s2, s3) in figure 2. (Junctions can be in one of a number of configurations (*stages*). The movements that have the green light during each stage are listed, where X : Y indicates the movement for vehicles travelling between node X and Y, through the junction node. By implication all movements not listed have a red light.)

node	stage	movements
s1	1	C:D,C:u1,C:s2,s2:D,s2:C,s2:u1
	2	D:C,D:u1,D:s2,u1:C,u1:D,u1:s2
s2	1	s1:E,s1:F,s1:s3,F:s3,F:s1,F:E
	2	s1:E,s1:F,s1:s3
	3	s3:s1,s3:E,s3:F
	4	E:s1,E:s3,E:F
s3	1	u1:A,u1:B,u1:s2,B:A,B:u1,B:s2
	2	A:u1,A:s2,A:B,s2:u1,s2:A,s2:B
	3	A:u1,A:s2,A:B

## Appendix B. Human-trained machine control neural network structure

Under HuTMaC control, each junction node in the network has a neural network associated with it and depending on which state description (loops or cells) is being used the number of input units and hidden units will be different. The precise set-up for each junction in the T-junction and Multi-junction networks is given in this appendix.

Tables 6 and 7 indicate which loops or cells are being used as inputs to which junction nodes of the T-junction and Multi-junction networks. The structure of the neural network at each node is also indicated.

**Table 6.** HuTMaC on the T-junction model operates in two modes using either the loop-based state or the cell-based state. (The top half of this table indicates which loops are used as inputs to the neural network operating at the junction node. The number of input units (*J*), hidden units (*H*) and output units (*K*) of the neural network are also shown.)

node	input loops	*J*	*H*	*K*
s1	all 11 loops	12	14	3

node	input cells	*J*	*H*	*K*
s1	all four cells	5	7	3

**Table 7.** HuTMaC on the Multi-junction model operates in two modes using either the loop-based or the cell-based state. (The top half of this table indicates which loops are used as inputs to the neural networks operating at the each junction node. The number of input units (*J*), hidden units (*H*) and output units (*K*) of the neural networks are also shown.)

node	input loops	*J*	*H*	*K*
s1	all 18 loops	21	24	2
s2	all 18 loops	21	24	4
s3	all 18 loops	21	24	3

node	input cells	*J*	*H*	*K*
s1	D:s1,C:s1,u1:s1,s3:u1,s2:s1	6	7	2
s2	s1:s2,E:s2,F:s2,s3:s2	5	7	4
s3	s1:u1,u1:s3,s2:s3,B:s3,A:s3	6	7	3

The number of patterns in the training data used to train each network are not shown in tables 6 and 7 because the value is the same for each row: *N*=1080 patterns. Following [23], the learning rate used in training was *η*=0.01.

## Appendix C. Relative performance of our subject

For consistency, the HuTMaC system presented in this paper was trained by a single human subject. It is interesting to consider how good the performance of our subject is relative to other humans. A version of the traffic control computer game was exhibited at the 2011 Royal Society Summer Science Exhibition in London. This week-long public engagement event was visited by 14 000 members of the public and 846 of them completed a 10 min game on the T-junction model, the average delay measured during the game was taken as the *score*. prior to the event our subject was also asked to play this game five times. Figure 9 shows a histogram of the difference between the score of visitors to the Royal Society Summer Science exhibition and our subject's average score. In other words, a negative score difference indicates that a player outperformed our subject and vice versa. Figure 9 indicates that our subject is a good but not exceptional player of the game.

## Appendix D. Detailed results table

Each of the simulation experiments described in §6 consisted of 10 independent, repeated runs. The data presented in §6 and table 1, in particular, are averaged over the 10 runs. Table 8 below presents the statistics of mean delay, *μ*(*θ*) and standard deviation over delay, *σ*(*θ*) for each individual run of the simulation experiments.

**Table 8.** Statistics of mean delay *μ*(*θ*) and standard deviation over delay *σ*(*θ*) for each of the individual 4 h simulation experiments described in §6. (A summary table presenting these data averaged over the columns is given in table 1.)

	T-junction								Multi-junction							
	MOVA		HuTMaC(loops)		HuTMaC(cells)		TD(cells)		SCOOT		HuTMaC(loops)		HuTMaC(cells)		TD(cells)	
run	*μ*(*θ*)(s)	*σ*(*θ*)(s)	*μ*(*θ*)(s)	*σ*(*θ*)(s)	*μ*(*θ*)(s)	*σ*(*θ*)(s)	*μ*(*θ*)(s)	*σ*(*θ*)(s)	*μ*(*θ*)(s)	*σ*(*θ*)(s)	*μ*(*θ*)(s)	*σ*(*θ*)(s)	*μ*(*θ*)(s)	*σ*(*θ*)(s)	*μ*(*θ*)(s)	*σ*(*θ*)(s)
1	27.00	65.17	24.46	39.52	23.11	42.56	18.09	34.40	50.25	35.69	37.54	37.82	26.81	21.49	25.65	19.36
2	19.64	35.77	22.04	39.07	21.98	39.69	18.82	35.56	51.97	43.37	32.09	26.42	27.89	21.70	25.54	19.35
3	24.39	50.67	18.17	27.78	25.63	45.18	16.42	26.36	48.69	34.77	32.13	26.73	26.73	19.05	26.06	19.53
4	25.20	60.89	20.59	34.17	22.10	35.69	16.32	23.96	46.81	35.90	34.66	28.91	26.98	19.85	25.13	19.57
5	23.50	47.61	24.46	42.71	21.72	34.54	17.84	30.14	50.64	39.28	33.84	29.89	27.15	20.14	24.76	19.26
6	27.86	57.32	20.54	35.24	22.59	38.30	19.06	33.10	50.23	37.82	32.16	27.74	27.59	20.35	25.60	20.33
7	24.52	46.67	22.11	40.19	24.53	41.76	18.41	31.72	50.14	37.42	31.65	27.45	26.98	20.15	25.76	19.50
8	24.89	47.38	19.92	31.09	22.93	40.73	18.41	34.38	49.27	35.66	34.76	32.15	27.35	19.88	25.15	19.02
9	21.40	38.40	18.94	31.41	24.56	42.60	19.14	33.94	49.85	39.13	32.88	33.55	27.15	20.01	25.52	18.42
10	21.13	36.69	23.90	38.64	22.44	39.65	20.91	39.11	49.78	39.23	31.43	26.34	27.36	20.34	25.22	19.89
mean	23.95	48.66	21.51	35.98	23.16	40.07	18.34	32.27	49.76	37.83	33.31	29.70	27.20	20.30	25.44	19.42

## Appendix E. Residual delays

The results on control algorithm performance presented in §6 were generated using the journey time data for all completed trips between source nodes and sink nodes in the network. When the simulation test terminates there will be some residual vehicles left in the network whose trips are not completed. Table 9 shows data on the residual delays in each of the simulation experiments described in §6.

**Table 9.** Comparing the mean delay over completed trips with mean delay over the small number of residual trips left uncompleted when the simulation terminates. (In all cases, the residual delay is lower, indicating that the inclusion of residual delays would bias overall delay to a lower value, as expected.)

	completed *μ*(*θ*)(s)	residual *μ*(*θ*)(s)
T-junction		
MOVA	23.95	−11.21
HuTMaC(loops)	21.51	−7.87
TD(cells)	23.16	−10.06
HuTMaC(ells)	18.34	−9.34
Multi-junction		
SCOOT	49.76	−3.05
HuTMaC(loops)	33.31	23.43
HuTMaC(cells)	25.44	−9.12
TD(cells)	27.20	−1.38

Note that the mean over residual delays may be negative as vehicles have only partially completed their trips and may have been travelling for less than the free-flow travel time (*δ*^(ff)^_*o*,*d*_) between nodes (see equation (2.2)).
